# Co-production of informal settlement health: a community based participatory research program for building healthy communities in urban informal settlements of Salvador, Brazil

**DOI:** 10.3389/fpubh.2026.1754353

**Published:** 2026-03-10

**Authors:** Hammed Mogaji, Lopez Yeimi Alexandra Alzate, Lívia Almeida Figuerêdo, Joao Henrique Araujo Virgens, Marie Agnes Aliaga, Hernan D. Argibay, Inajara Salles, Andreane Pereira Moreira, Terezinha de Jesus Lima e Silva, Suzana Cristina dos Santos, Rita Batista, Elizete Cardoso, Elenilda Cardoso Neves Santos, Edlane Leal dos Santos, Edlana Rodrigues dos Santos, Thiago da Mata Barreto, Thais Auxiliadora dos Santos Mattos, Nivison Nery, Jaqueline Cruz, Ianei Carneiro, Ricardo Lustosa, Victoria C. Dedavid Ferreira, Mitermayer Reis, Albert I. Ko, Federico Costa, Mike Begon, Hussein Khalil

**Affiliations:** 1 Instituto Gonçalo Moniz, Fundação Oswaldo Cruz, Ministério da Saúde, Salvador, Brazil; 2 Instituto de Saúde Coletiva, Universidade Federal da Bahia (UFBA), Salvador, Brazil; 3 Department of Epidemiology of Microbial Diseases, Yale School of Public Health, New Haven, CT, United States; 4 Programa de Pós Graduação em Educação e Contemporaneidade, Universidade do Estado da Bahia, Salvador, Bahia, Brazil; 5 Association of Residents of Alto do Cabrito Salvador, Salvador, Brazil; 6 Cia de Arte Cultural E ao Quadrado, Salvador, Brazil; 7 Associação Emília Machado Bahia, Salvador, Brazil; 8 Programa de Pós Graduação em Biodiversidade e Evolução, Instituto de Biologia, Universidade Federal da Bahia, Rua Barão de Jeremoabo, Ondina, Salvador, Bahia, Brazil; 9 Department of Preventive Veterinary Medicine and Animal Production, Escola de Medicina Veterinária e Zootecnia, Universidade Federal da Bahia (UFBA), Salvador, Brazil; 10 Faculdade de Medicina da Bahia, Universidade Federal da Bahia, Salvador, Brazil; 11 Department of Evolution, Ecology and Behaviour, University of Liverpool, Liverpool, United Kingdom; 12 Department of Wildlife, Fish, and Environmental Studies, Swedish University of Agricultural Sciences, Umeå, Sweden

**Keywords:** Brazil, community-based participatory action research, health inequities, health justice, Popular Health Education, urban slums

## Abstract

**Introduction:**

More than 15% of Brazil's urban population lives in slums characterized by limited access to essential urban services, heightened vulnerability to infectious pathogens and environmental hazards, and deprivation of citizenship rights. These conditions exacerbate social inequality, perpetuate cycles of poverty, and fuel violence, underscoring the urgent need for sustainable interventions.

**Methods:**

Following a social justice framework, we developed a community development program rooted in participatory research methods and popular health education to foster collaboration between university researchers and communities. The aim was to identify priorities and co-create locally driven, cost-effective, and sustainable solutions. This article describes our ongoing project in three prominent urban slums of Salvador, Brazil (Alto do Cabrito, Pau da Lima and Marechal Rondon), detailing the methodologies employed, activities initiated, and interventions developed.

**Results:**

We conducted ethnographic, eco-epidemiological, and collaborative mapping surveys to contextualize diverse health and well-being challenges. Furthermore, we organized consultative and socialization events with dynamic community groups and identified local priorities, leading to the design of thirteen interventions targeting citizenship rights, social cohesion, environmental restoration, waste management, and unemployment.

**Discussion:**

Here, we described how our interdisciplinary approach leveraged social capital and fostered inter-sectoral partnerships to empower marginalized urban communities towards addressing their health and environmental challenges through sustainable, locally tailored solutions. While the program has strengthened community trust, facilitated partnerships, and achieved notable environmental improvements, further evaluation is needed to assess the long-term impacts of these interventions on broader social health determinants.

## Introduction

Over a billion people reside in informal settlements within urban areas (1). These settlements are characterized by socioeconomic, environmental, and infrastructural deficiencies that impact resident wellbeing, safety, social and political inclusion, and livelihood opportunities (2).

In Brazil, social and economic inequalities (3, 4) have resulted in the establishment and expansion of urban informal settlements, commonly referred to as “*favelas*” (5). More than 15% of the Brazilian urban population resides in *favelas*, where they have limited economic opportunities, insufficient access to basic urban services, including clean water and sanitation (6), often living in substandard and overcrowded domiciles, subject to seasonal flooding and erosion risks, which increases the exposure to pathogens whose transmission is favored by such poor socio-economic and environmental conditions (7–10). The socioeconomic and health disparities, and in some cases, violence in informal settlements are closely linked to feelings of deprivation of citizenship rights and privileges (11). Over the last two decades, our team has been engaged in the study of infectious disease in residents of urban informal settlements in the Brazilian city of Salvador, Bahia (7–10). We here describe a participatory program, which has evolved within our overall study to address health and wellbeing more generally and their social, demographic, and environmental determinants.

Initially the focus, led by medical scientists, was on the epidemiology of leptospirosis, a neglected but important human bacterial disease transmitted from the urine of a zoonotic reservoir host, predominantly the brown rat, *Rattus norvegicus* (9, 10, 12–14). Subsequently, our scope broadened to encompass ecological studies of leptospirosis in its *R. norvegicus* reservoir (15), and in the wider environment (16), and to other infectious diseases, including dengue (17), zika (8), and SARS-CoV2 (18). Recently, the research program broadened further to integrate social scientists and promote community engagement and participation (19, 20), and address wellbeing more broadly.

During this program, our studies have highlighted the need for investments in environmental improvements, basic urban services, and enabling residents to protect themselves and their domiciles from sources of environmental contamination (21). However, such transformations demand significant government investments, which, in practical terms, may not materialize within an expected timeframe. Instances of political neglect and disparities in the allocation of infrastructural resources to informal settlement populations are not uncommon (22), leading often to sporadic and localized interventions that lack coordination, and which are therefore unsustainable. Sustainability is further undermined by interventions often being short-term and poorly adapted to the local context, which raises issues of acceptability, appropriateness, and feasibility (23).

These considerations, and the growing complexities associated with urbanization and inequality globally, argue for the need, far more widely acknowledged in the social than in the natural sciences (24–29), for teams such as ours to adopt approaches that fully involve community residents in the creation and delivery of interventions tailored to the specific context of informal settlement areas. Our on-going program, described in this article, adapts and builds on the principles of Community-Based Participatory Research (CPBR) and Popular Health Education (27, 28), with the primary aim of driving action and facilitating social change based on local priorities (29, 30). This necessitates a recognition of professional researchers' social obligation and commitment to research processes that can drive sustainable change (24), predicated on the premise that *co-construction* enables deeper engagement, which may be key not only to health promotion, but also to social transformation and emancipation in these informal settlements (25, 26). Co-construction is defined here as the participatory and iterative process through which professionals and community members jointly design and implement activities and interventions.

We identified several key attributes for the success of this approach, including successful blurring of lines between researchers as neutral and objective observers and research participants, minimizing power imbalances, approaching health and wellbeing with a holistic and community-based perspectives, and conducting research in partnership with communities to achieve common goals: sustainable, long-lasting positive outcomes that extend beyond the duration of the research (29). Our aim is to address the paucity of CBPR approaches necessary for improving health outcomes among informal settlement populations (22, 31).

We worked with residents and other stakeholders in three communities that are superficially similar, but have contrasting histories and social characteristics, leading to three unique CBPR experiences. These included a range of participation dynamics and community empowerment activities and outcomes, leading to cross-fertilization arising from the interaction among communities and our interdisciplinary research team. Nevertheless, a common aim in the three communities was to address simultaneously multiple problems of health and wellbeing (a range of infectious diseases, rat infestation, food security, and environmental and community wellbeing) and evaluate multiple outcomes related to the participatory and organizational processes, stakeholder satisfaction, and individual, community, animal, and environmental health.

Below, we describe the processes and interventions achieved in the initial phase of our program. In doing so, we draw an important distinction between what we describe as “activities” and “interventions.” Indeed, the difference between activities and interventions is a key characteristic of the approach described here. We define an activity as an action that is initiated by the external research team (i.e., *not* by the community), which has the proximate aim of facilitating community engagement or empowerment—defined here as strengthening community capacities to identify and communicate health challenges, actively participate in the co-construction of solutions, and contribute to their evaluation. Each activity can thus be assessed primarily by its success in promoting meaningful engagement and empowerment, as well as by its ability to generate, through sustained interactions between the research team and the community, one or more context-specific interventions. An “intervention,” then, is defined as an action initiated by an empowered and engaged community in collaboration with the external research team, with the aim of improving the health and/or the wellbeing of the community and can thus be assessed by its success in doing so in the short and/or the long term.

We first provide the health context of the study area and described the range of *activities* undertaken. This is “what was done” and might therefore be considered equivalent to our “Methods.” We then describe the proposed *interventions* generated which may be considered as our “Results.” Finally, we discuss several key lessons emerging from reflections on the process leading from activities to interventions, including how they differ from the conventional solutions health experts might have suggested, and how their success is currently being assessed, and will be documented in subsequent articles.

## Methods

### Study area, population, and context

**Figure 1:**
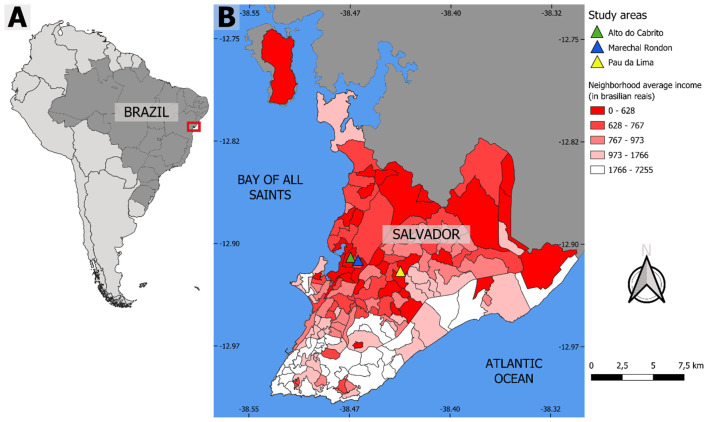
Map of Brazil **(A)** and Salvador showing the location of the three marginalized communities where we work **(B)**. The neighborhoods of Salvador are classified by the mean income of the head of the household to show the rent spatial segregation observed in the city. The maps are not copyrighted as they were created by the authors using QGIS 2.18 software [Open Source Geospatial Foundation (OSGeo), California, USA]. The boundary polygons of Brazil, Salvador and the production of the Salvador income map were downloaded from open and publicly accessible base of IBGE—Instituto Brasileiro de Geografia e Estatistica, on the Geosciences platform which can be accessed at https://www.ibge.gov.br/geociencias/downloads-geociencias.html. The WorldView-3 May 2017 satellite image was also used to digitize the study area. The image was acquired by the research project/Instituto Gonçalo de Moniz—IGM—Fiocruz Bahia from the company Satmap (Salvador, Brazil), with disclosure permitted referencing the Copyrights of DigitalGlobe images.

Our program was implemented in Salvador, Bahia, Northeastern Brazil, a city with approximately 2.5 million inhabitants according to the 2022 Census conducted by the Brazilian Institute of Geography and Statistics (IBGE) (6). In Salvador, 45% of households live in informal settlements (6), and the city has about 7% of all urban informal settlement residents in the country (32). Most of our previous studies have been implemented in the Pau da Lima community (8, 9, 18), and more recently in four other similar marginalized peri-urban settlements including two considered here, namely Marechal Rondon and Alto Do Cabrito (10, 19). All these communities are extremely vulnerable to flooding and soil erosion due to their open sewer systems and their topographic properties, including steep valleys (Figure 1). In these communities, most residents lack legal titles to their homes, and the median household per capita income is US$2.32/day (18). Majority were either unemployed or informally employed as artisans, in domestic and construction work (18, 19, 21). During the COVID-19 pandemic, economic conditions pushed more than two-thirds of the population into hunger and close to 80% into unemployment (10, 33). The communities differ in age of establishment, yet variation in socioeconomic and environmental conditions appears to be larger within than between the communities (10, 33). Public services, including transportation, sewage systems, and primary health centers, are generally accessible to residents and are located around the slum communities within walkable distance. In Brazil, these services are typically delivered through decentralized municipal systems, with primary health care provided through local health units linked to the Unified Health System (SUS). Within each community, heterogeneous local structures exist, including neighborhood associations, cultural associations, youth groups, and religious organizations, which serve as important channels for communication and community mobilization. While neighborhood associations often engage with local government structures, cultural and youth associations were specifically engaged in this study because they have minimal political affiliation and provided neutral platforms for community participation. The study areas ranged between 0.07 and 0.09 km^2^ in extent within each community, with approximately 3,000 participating residents living in over 1,000 households (see Table 1 for additional demographic and contextual information).

**Table 1:** Baseline profile of study communities included in the interdisciplinary project (November 2021).

**Variables**	**Study communities**		
	**Pau da lima**	**Marechal Rondon**	**Alto Do Cabrito**
Study population size	1,834	816^c^	93
Number of households	833	463^c^	51
Female population	58.1%	60.5%	61.3%
Median age (in years)	32	35.0	34.0
Predominant race-black/brown	94.3%	90.5%	90.3%
Literacy level^a^	34.5%	39.4%	46.2%
Food insecurity^b^	45.2%	67.2%	89.2%
Unemployment^c^	61.4%	53.3%	48.8%
Seroprevalence of SARS-CoV-2	75.9%	91.2%	89.1%
Seroprevalence of leptospirosis	10.2%	12%	7.6 %
Seroprevalence of Chikungunya	44.2%	40.1%	46.7%
Seroprevalence of Dengue fever^d^	61%	82.9%	81.5%
Physical health score^e^	53	48	46
Mental health score^e^	51	46	47

^a^Than 6 years of formal education.

^b^Responses were from head of households or another eligible representative.

^c^Unemployment was assessed for adult participants (18 years and above).

^d^Prevalence among residents under 20 years of age.

^e^Reference threshold for good health score is 50 (21).

### Program design

**Figure 2:**
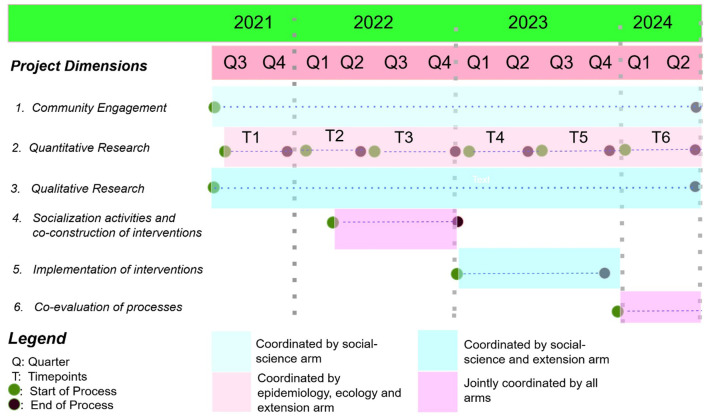
Schematic diagram of the project design.

Our program includes the following dimensions: (i) community engagement (events and activities) to facilitate the entry and participation of community residents and other stakeholders, (ii) quantitative, eco-epidemiological research to estimate exposures of residents and domestic animals to disease reservoirs and vectors, as well as collaborative environmental and perception mapping to provide accurate geo-location of the problems identified by the communities. Additionally, the project includes (iii) qualitative research and ethnographic studies to understand the processes of participation and the perceptions of residents about the various processes of research, (iv) socialization activities for co-construction interventions, (v) dialogue and cooperation with local agencies (public and private) for the implementation and possibility of scaling up the proposed interventions, and finally (vi) co-evaluation of research processes with communities, which includes quantitative and qualitative parameters (Figure 2).

### Team composition and role

The program had three interlinked arms: (i) social sciences, (ii) ecology and epidemiology (eco-epidemiology), and (iii) collaborative mapping and extension. Briefly, the social sciences arm was coordinated by a team of social scientists, educators and psychologists. They organized socialization activities including engagement within and between teams and residents, performed ethnographic surveys, and subsequent curation and dissemination of research findings. The eco-epidemiological arm was coordinated by a team of epidemiologists, ecologists, statisticians, database managers and laboratory technicians. They performed and supervised surveys between 01 November 2021 and November 2024 to collect demographic, socioeconomic, and environmental data as well as estimate resident and domestic animal exposure to infectious diseases. The collaborative mapping and extension arm conducted mapping activities, focusing on resident perceptions of, and attitudes regarding, their direct (peri)domestic environment and community, including areas and environmental features they prioritize for interventions. This complements the qualitative and survey-based activities of the two other project arms. Additionally, this arm led capacity-development initiatives within the community through institutional collaborations and engagement with governmental and other third-party organizations.

While each project arm took the lead on one or more of the project dimensions, the participatory dimension and the evaluation of all interventions and the process were jointly led. More importantly, even for “disciplinary” dimensions, continuous dialogue among different teams and the exchange of experiences, ideas, and mutual curiosity ensured that our project constantly evolved and interdisciplinarity increased. This was exemplified by the co-construction of novel study tools, generation of ideas, and subsequent implementation of collaborative activities among the arms.

### Community engagements

The primary strategy for engaging residents across the three study areas in the design and implementation of interventions was through establishment of community working groups. The social sciences team facilitated community mobilization processes and supported the formation of these groups, which served as platforms for identifying challenges and co-designing locally appropriate interventions. Attempts were made to include representatives from local health facilities and municipal government agencies in the working groups; however, participation from these stakeholders was limited despite invitations. The meeting dynamics followed popular education methodologies and a problem-solving approach, allowing residents to decide on discussion topics and actions to be undertaken in their communities. This approach is grounded in the traditions of Popular Education and Community-Based Participatory Research as developed in Latin American public health scholarship (28, 34). Outputs from these discussions were documented as meeting notes or minutes and subsequently used to guide follow-up discussions and implementation activities in later meetings. Some interventions were supported through written agreements and institutional partnerships with public and private agencies. However, most interventions were implemented through informal commitments grounded in trust, shared responsibilities, and ongoing collaboration among stakeholders. Table 2 provides an overview of the community groups, including meeting location, frequency, and format, as well as key themes discussed in those meetings.

**Table 2:** Profile of community groups across the study communities included in the interdisciplinary project (November 2021).

**Variables**	**Study communities**		
	**Pau da lima**	**Marechal Rondon**	**Alto Do Cabrito**
Were there existing community groups?	Yes^a^	Yes^b^	Yes^c^
Make-up of group	Mostly women and children and researchers	Community leaders, youths and researchers	Women (community leaders, residents, youths) and researchers
Size of group	Dynamic^d^	Dynamic^d^	Dynamic^d^
How we contextualize needs	Open questions in the sero-survey (21) and discussions held in the Freirean tent	Open questions in the sero-survey (21) and conversation circles with young people and leaders based on data from previous research and other problematization	Open questions in the sero-survey (21) and conversation circles with community leaders, youths and leverages on previous research in the locality
How group members were invited	Door-to-door visits to residents. Delivery of printed invitations, and wall posters in the community	Contact with AEMBA and recruitment at the local school	Contact with E^2^ and a snow-ball sampling approach among community leaders/stakeholders
Meeting venue	A newly created open tent by the team called a “Freirean Tent”	AEMBA and Community School	E^2^ Theater Cultural Center
Frequency of meetings	Fortnightly	Weekly	Bi-weekly
Format of meetings	Physical	Physical and virtual	Physical and Virtual
Themes of group discussions	Community needs, problems and aspirations and definition of interventions	Community needs, problems and aspirations and definition of interventions	Community needs, problems and decision on priorities in interventions

^a^Yes, but in the serosurvey questionnaire, people indicated that they did not feel represented by them.

^b^Emília Machado Association (AEMBA).

^c^E^2^ Theater Cultural Center.

^d^Participation is open, hence the groups are heterogeneous and dynamic in nature.

For instance, the Pau da Lima community is very heterogeneous, characterized by its diverse composition of four valleys. We decided to work in the first valley “Baixa de Santa Rita,” where social vulnerability is highest and, as unpublished feedback sero-survey questionnaires revealed, residents did not perceive that they were adequately represented by neighborhood associations and leaders. Additionally, there was a notable absence of leisure activities or avenues for community participation reported by individuals in the area. Hence the team conducted door-to-door visits and discussions to contextualize needs, resulting in a greater participation of women who arrived with their young children to join group discussions. The team established an open tent called the “Freirean Tent,” serving as the meeting venue every fortnight. To promote engagement and inclusive dialogue, we employed methodologies rooted in popular education and participatory research, including social cartography (28, 34–38). This approach enabled the identification of community needs, challenges, and aspirations for a better future. Interestingly, the involvement of children under 12 years old was unplanned initially, but mothers arrived with their children seeking participation in the sessions. Consequently, we adapted by organizing parallel recreational, sports, and artistic activities for the children, alongside the main discussions. These gatherings facilitated dynamic conversations on potential intervention strategies that could be prioritized to address community issues (Table 2).

In selecting the participatory group for the Alto do Cabrito community, we collaborated with the “Cia de Arte Cultural E ao Quadrado” (E^2^), an artistic and cultural association. The pre-existing relationship, established during research conducted from 2017 to 2019 (10), fostered trust between the E^2^ leaders and our project team, facilitating continued collaboration (Table 2).

In Marechal Rondon, like Alto do Cabrito, there existed a prominent Community Association known as “Associação Emília Machado Bahia” (AEMBA), which we had also engaged with in previous projects. AEMBA focuses primarily on youth capacity-building initiatives. To understand the community's needs, we conducted key informant interviews with local leaders and drew upon knowledge gained from our previous research in the area. Youth recruitment for group discussions took place at AEMBA, and meetings became more frequent with the initiation of the current project. The discussions led to collaborative mapping exercises and the identification of key themes by young participants from AEMBA and Colegio Germano Machado, a school in the community, which subsequently informed action plans (Table 2).

### Research activities of the eco-epidemiological, and collaborative mapping arms of the project

The eco-epidemiological arm conducted surveys to estimate zoonotic and mosquito-borne pathogen burdens and associated risk factors in the study areas. For individuals above the age of 5, bi-annual serological surveys were conducted at households across study communities, involving the collection of blood, nasal swabs, and saliva (from humans). For domestic animals (e.g., dogs, cats, chickens), blood, oral/rectal swabs and feces were collected to assess health conditions and zoonotic infections. Ecological assessment involving seasonal environmental surveys using track-plates and oviposition traps were used to estimate rodent and mosquito populations, respectively (39, 40). Remote sensing techniques were also used to assess the presence and extent of open sewers, drainage, trash accumulation points, and land cover/land use. Water from open sewers and puddles, as well as soil from randomly selected points, were collected to quantify pathogen loads in the environment. These data were complimented by those provided by the mapping and extension team, which coordinated all project-related mapping activities, focusing environmental features that were identified by residents as points of strength or which needed improvement. The team also emphasized community empowerment through the establishment of cultural, artistic, and communication programs.

### Socialization of results and co-construction of interventions

All socialization activities (communicating and reflecting on the current state of data-based knowledge to the broader community) happened after the first 12 months of initiating the project, which coincided with the period where results generated from established working groups, and eco-epidemiological surveys had been analyzed for dissemination at community meetings. The process of socialization was different in each community. The Alto Do Cabrito community served as the pilot site for socialization of results, which involved several workshops and engagements between the team and the residents over a period of 4 months. The workshops provided a platform for our team to share their earlier research findings, enabling interdisciplinary discussions among experts in epidemiology, ecology, extension, and social sciences. The workshops involved community debates based on a video synthesis of research results, mediated by field researchers., and focused on community feedback regarding how they perceived the implications of previous results for health interventions in the community.

In Marechal Rondon, the socialization was not limited to meetings, but also a series of engagements between researchers and youths through training activities, field tours and mapping of identified vulnerabilities. The pre-existing interest of the youths in activities related to environmental health, vector and reservoir ecology allowed the exchange of knowledge and research findings to be more fluid and constant throughout the project.

In Pau da Lima, the longer-term engagement of the broader research team with the community led to the socialization process taking on a distinct dynamic. Initially, the project teams proposed a review of published articles from research studies conducted in Pau da Lima over the last two decades. All teams, including community mobilizers, participated in this process, with the aim of synthesizing these studies and producing written or online materials. However, as the article summaries were being finalized, a sewerage improvement project commenced, disrupting our regular fortnightly meetings in the Freirean tents. During subsequent meetings, we therefore focused on those research findings related to sewerage improvements and health in discussing the design of interventions in the community.

## Results

This section outlines the objectives, key activities, stakeholders, and expected outcomes of the interventions implemented in the three communities.

### Alto-Do Cabrito community

A total of five interventions were developed in Alto do Cabrito (Table 3). The first intervention proposed by the community group involved creating a memorial to document the community's history. This initiative aimed to challenge the prevailing narratives about the neighborhood, which often focused on themes of violence and drug trafficking. The memorial project included capturing audiovisual narratives from residents of the neighborhood. Group members organized themselves to conduct more than 20 interviews with individuals recognized for their significant roles in the community, whether as religious figures, cultural influencers, or social leaders. Beyond sharing these videos on a dedicated website, the collaborative efforts have been showcased in a community exhibition. The memorial project has facilitated a re-examination of the neighborhoods' narratives, highlighting its identity as a peripheral area while also exploring the connection between present-day health issues and collective memory.

**Table 3:** List of interventions developed in Alto do Cabrito community.

**Intervention**	**Aim**	**Activities**	**Actors**	**Frequency**	**Immediate impact**	**Long term impact**
Community memorial	Preserve the life story and narratives of residents, their memories, experiences and knowledge about life and health in the territory	Recording interviews and audiovisual production of narratives condensed into 3 documentaries and development of an open repository (i.e., webpage)	Residents (leaders and people of reference in the community)	Continuous	Encouragement and strengthening of bonds and sense of belonging within and between neighborhoods, contributing to countering stigmatizing and prejudiced hegemonic narratives about the territory.	Strengthen cohesion among community group members and improve intergenerational relationships. Contribute to the construction of health based on the perception of memory as a human experience.
Community health fair	Promote integration between communities. Promote awareness about community working groups. Promote health in an expanded way and collaboration between different social, health, artistic and cultural entities	Socialization of preliminary research results, thematic dialogues, provision of health services and carrying out integrative activities (cultural, artistic, poetic displays)	Residents and researchers	Point	Encouraging engagement and building bonds (between residents, communities and research teams); Expanding the communal conception of health	Strengthening autonomy and self-management dynamics; strengthening partnerships with other entities
Dique Verde Vivo Project	Contribute to the socio-environmental recovery of Dique do Cabrito	Providing awareness about environmental management; Assisting with claims from public authorities; Implementing environmental and artistic interventions (cleaning the surrounding area and the waters of the Dique)	Community residents and researchers, LIMPURB, SEMOP, and INEMA	Continuous	Improve the aesthetics of the neighborhood; Reducing exposure to disease vectors; Promoting care for the environment; Valuing art and local artists and strengthening articulation between residents in organizing the event; Integration between communities (Marechal Rondon and Alto do Cabrito)	Improve environmental conditions and quality of life for the population living around the Dique, as well as strengthening community cohesion
Bota Fora	Promote the removal of unusable waste materials from abandoned spaces	Removal of unusable waste from abandoned spaces with the support of residents and subsequent collection by the public waste agency.	Residents mostly youths, researchers and LIMPURB	Point	Improve the aesthetics of the neighborhood; Reducing breeding sites for rodents and vectors; Promoting better air quality and improving overall quality of life.	Improved environmental conditions as measured by reduced incidence of vectors, rodents and associated diseases; improved; Improved unity among community groups
Artistic paintings (complementary to Bota Fora)	Redevelopment of degraded or abandoned spaces	Mapping of abandoned or degraded spaces and transformation of such places to social hubs using artistic works	Residents mostly youths	Bi-monthly	Improve the aesthetics of the neighborhood; Promoting the prevention of using renovated spaces as dumping sites	Promote interest in renovating abandoned spaces with artistic work. Improved environmental conditions as measured by reduced incidence of vectors, rodents and associated diseases. Improved unity among community groups

The second intervention involved organizing a health fair aimed at fostering collaboration among various social, health, artistic, and cultural entities. In partnership with the Federal University of Bahia (UFBA), planning meetings were held to develop dynamic organizational strategies, plan activities, and secure funding. In addition to health professionals, local artists were also involved, collaborating with residents to develop proposals aligned with the broader health perspective for inclusion in the fair. The health fair venue featured a dissemination tent where the research's most significant findings were showcased through interactive panels and discussion circles (Table 3).

The third intervention was the “Dique Verde Vivo Project” (literal translation “Living Green Dike project”). It was initiated by the community group, with the aim of restoring the Dique do Cabrito, an artificial lake that runs through the community's territory and is linked to the history of socio-cultural development of the neighborhood. The idea of restoring the lake emerged from the people interviewed for the memorial, as their narratives highlighted the importance of the Dique for the community. A number of health and socio-environmental issues linked to the Dique were also mentioned during the socialization of the results. The Living Green Dike project encompasses a range of activities including cleaning of the lake, educational campaigns aimed at raising community awareness for cleaning the lake, tree planting around the lake, a collective action to assemble a “Christmas tree” using recyclable materials, and the creation of a poetry wall. The latter is a cultural activity featuring presentations by around 130 local artists, facilitating the exchange of existing artistic potential within the neighborhood. To carry out these activities, this project has actively sought partnerships from governmental agencies responsible for (i) refuse collection i.e., LIMPURB- Empresa de Limpeza Urbana de Salvador (literal translation “Urban Cleaning Company of Salvador”) (ii) SEMOP—Secretaria Municipal de Ordem Publica, (literal translation “Municipal Secretariat of Public Order”) and (iii) INEMA- Instituto Do Meio Ambiente E Recursos Hidricos (literal translation “Institute of the Environment and Water Resources”) to plan and execute actions (Table 3).

Fourth, the “Bota Fora” intervention (literal meaning: “to kick out”) aims to improve environmental quality, repel rats and mosquitoes, and enhance aesthetics of neighborhoods by removing unusable waste materials from abandoned spaces. With the active involvement of residents, particularly youths, researchers, and the public waste agency (LIMPURB), this intervention was designed to clean up abandoned areas and dispose of waste properly. By clearing these spaces, the intervention seeks to reduce breeding sites for rodents and disease vectors, thereby improving environmental conditions and air quality. The removal of waste also contributes to a better overall quality of life for residents by creating cleaner and more pleasant surroundings. Additionally, this also aims to improve unity among community groups as they collaborate toward a common goal of enhancing the neighborhoods' cleanliness (Table 3).

Finally, the fifth intervention is complementary to “Bota Fora” and involves redevelopment of degraded or abandoned spaces within the community using artistic paintings. Through collaborative efforts primarily involving youths, these spaces are identified through mapping exercises and transformed into vibrant social hubs adorned with artistic works. The initiative aims to improve the aesthetics of the neighborhood and deter residents from using these spaces as dumping sites. Through the creation of visually appealing areas, residents are encouraged to take pride in and maintain these spaces, fostering a sense of ownership and community pride. Additionally, this initiative contributes to improved environmental conditions by reducing the incidence of vectors, rodents, and associated diseases, thereby enhancing overall public health. As the community groups work together toward revitalizing their shared spaces, this initiative will also create a more vibrant, inclusive, and collaborative community environment (Table 3).

### Marechal Rondon community

In Marechal Rondon, as the group was primarily made up of young people, the first set of interventions was related to the development of community and university courses and workshops to prepare young people for the job market, considering the greater difficulty in accessing jobs for young people living in peripheries (41). These activities include courses in English and French languages, computer studies, robotics, theater, singing, guitar, dance, college entrance exam preparation classes, entrepreneurship, first-aid and graffiti. Some of the young people who participated in these courses were available to share what they learned with other young people in the community after the project ended and were recognized as teachers, bringing them satisfaction due to the feedback they have received and the difference they are making (see Table 4 for details).

**Table 4:** List of Interventions developed in Marechal Rondon community.

**Intervention**	**Aim**	**Activities**	**Actors**	**Frequency**	**Immediate impact**	**Long term impact**
Community-run courses	Developing skills in various areas of knowledge with teachers from the community itself	Engage youths in courses such as informatics, photography, arts, English and music.	Young teachers from the community	Bi-annually	Improved skill sets for job opportunities; Enhanced capacity and improved self-esteem for young community teachers; Improved valuation for teaching profession; strengthened association within the community	Established network of professional teachers; Increased interest of young teachers and students in advanced education; Enhanced engagements amongst youths for sustainability.
University-run courses	Developing skills in various areas of knowledge with teachers from the university (research group)	Engage youths in language classes (French, English), computer courses, graffiti and collaborative mapping exercises	Researchers from the universities and youths in the community	Bi-annually	Improved skill sets for job opportunities; improved capacity and self-esteem for youths to interact with global audiences	Established network between youths and researchers; Increased interest of youths in advanced education; Enhanced capacity and engagements amongst youths for sustainability.
Startup social CommuniTech	Empower youth from communities to take leadership in digital culture and engage in discussions about inequalities.	Creating and hosting podcasts; Offering computer and technology courses; Producing audiovisual content; Participating in conferences and seminars	Youths from community	Monthly	Facilitating communication on technology and inequality topics relevant to peripheral youth, presented in accessible language; Inspiring other young people to take initiative and become involved in social startups.	Establishing sustainable practices and fostering new interventions that benefit future generations
Periphery in Every Corner (PETOC)	Promote citizenship and guarantee the right to the city and art (access to desired and unknown spaces)	Organize visits to locations selected by the youth, such as museums, tourist attractions, universities, parks, squares, and more. Facilitate artistic performances by the youth in these spaces; Conduct discussions before and after the visits to reflect on the activities and the spaces explored.	Youths from community and researchers	Bi-monthly	Increased awareness of their surroundings within typically exclusionary spaces; Strengthened interpersonal relationships within the group; Active participation in activities connected to local associations; Encouragement of artistic expression and the use of public and university spaces.	Cultivation of critically aware individuals who challenge inequalities and exclusionary spaces; Sustained commitment to social causes and advocacy
Crochet workshop	To foster social engagement and strengthen intergenerational dialogue among community members through a creative and inclusive activity.	To create a platform where women from different backgrounds can gather weekly to crochet while discussing social issues relevant to their living conditions and the challenges of their territory	Community women	Weekly	Establishment of a supportive group where participants can engage in creative activities; Increased awareness and dialogue around social issues affecting the community; Initial connections and mutual understanding between participants of different generations.	Strengthened intergenerational relationships and a more cohesive community; Empowerment of women to advocate for improvements in their living conditions; Sustainable development of a community-driven initiative addressing social issues collaboratively.
Bota Fora	Promote the removal of unusable waste materials from abandoned spaces	Removal of unusable waste from abandoned spaces with the support of residents and subsequent collection by the public waste agency.	Residents mostly youths, researchers and LIMPURB	Point	Improve the aesthetics of the neighborhood; Reducing breeding sites for rodents and vectors; Promoting better air quality and improving overall quality of life.	Improved environmental conditions as measured by reduced incidence of vectors, rodents and associated diseases; improved; Improved unity among community groups

The second intervention was “Periferia em Todos os Cantos” (PETOC) project (literal meaning: Periphery in Every Corner), an initiative that enabled young people to occupy spaces in the city of Salvador that many of them had not previously visited and, in some cases, never considered they could access. The PETOC project promoted artistic performances in various locations in the city, such as the Federal University of Bahia and historical and touristic neighborhoods such as Barra and Pelourinho (Table 4).

The third intervention, like the memorial intervention in Alto Do Cabrito, was the creation of a podcast, by young residents, to identify and address problems in the community and those identified by the community groups. This initiative seeks to recover memories and expand the possibilities for dialogue in the community on contemporary issues, particularly those related to the environment, inequalities, social empowerment and innovations.

The fourth intervention was a crochet workshop, which aimed to bring together a different audience from those who already attended the group and to stimulate intergenerational dialogue. Through this initiative, a group of women was formed who began to meet weekly to crochet and discuss social issues related to their living conditions and the territory in which they live (Table 4).

Finally, the fifth intervention was the “Bota-for a,” similar to that developed in Alto do Cabrito, with the two community groups coming together to carry out the activity in partnership. However, in Marechal Rondon, there were instances where the researchers interacted extensively with residents and shared information on how to produce homemade repellents to prevent mosquitoes that transmit dengue fever (Table 4).

### Pau Da Lima community

A total of four interventions were developed in Pau da Lima. The covering of sewage outflows was implemented by the municipal project and not an outcome of our project. However, the community residents and researchers joined the agency in monitoring and improving the coverage of the intervention. The other three interventions were designed from the “Freirian tent” during our socialization activity, where residents preferred interventions aimed at strengthening community spaces and social bonds beyond the project's duration (Table 5).

**Table 5:** List of interventions developed in Pau da Lima community.

**Intervention**	**Aim**	**Activities**	**Actors**	**Frequency**	**Immediate impact**	**Long term impact**
Coverage sewage channels^*^	Cover sewage channels that pollute the Trobogy and Coroado tributaries of the Jaguaribe River	We followed the project, encouraging dialogue between residents and those responsible for the work with the aim of maximizing its scale and benefits	Municipal government agencies.	Point	Improved health conditions and the dynamics of community coexistence.	Improved quality of life
Community spaces	Improving the neighborhoods' infrastructure and expanding access to public services	Construction, and management of parks, playing square, daycare center and family health unit (collectively regarded as eco-social hubs)	Community residents and researchers	Continuous	Improved access to basic services such as parks, playing square, daycare center and family health unit (collectively regarded as eco-social hubs),	Improved quality of life
Sports, creative and professional workshops	Promote personal and sociocultural development activities among children and adults within and between communities.	Sporting, recreational and other professional activities targeting children and young people, such as boxing classes, theater and photography workshops	Community residents and researchers	Continuous	Promote strengthening of bonds among children and adults within and between communities; Develop creative and professional capabilities of people in the community; Recognize and discuss the health problems involved in Baixa da Santa Rita	Promote integration among children (for example those with disabilities) and challenge existing social barriers
Community garden and kitchen	Promote activities to combat food insecurity and unemployment	Construction of a community garden and kitchen; Establishment of dialogues on food security and income generation	Community residents and researchers	Continuous	Improve food security and relationship with the environment; Improve employment and income conditions, based on practices that encourage solidarity and the construction of citizenship.	Improved quality of life; Improved Food security; Improved income

^*^Interventions were implemented by the municipal government outside the project and were only monitored by the community residents and researchers.

The first of these interventions focused on the development of spaces that serve both ecological and social functions. This initiative aimed at improving the overall quality of life within the neighborhood and will involve the construction and management of various amenities, including parks, playgrounds, daycare centers, and family health units (Table 5).

The second group of interventions was the development of sporting, recreational and other professional activities targeting children, young people and adults, including boxing, theater, photography, crafts and makeup workshops to promote integration among the community's residents and to challenge existing social barriers, as well as courses aimed at expanding the possibilities for young people to enter the job market. The makeup course (coordinated by a resident), in addition to sharing beauty techniques, was created to be a space to discuss self-care and socially imposed beauty standards. The crafts course (also coordinated by a resident) was attended by adults and children, sharing different techniques, mainly using recyclable materials to produce art (Table 5).

The third intervention was a community garden and kitchen, which was designed for young people and adults, emphasizing alternatives for income generation while addressing food insecurity issues and improving nutrition for residents facing financial constraints. It involves the construction of the community garden and kitchen and organizing regular dialogues on food security and income generation in the community (Table 5).

## Discussion

In this article, we have described our interdisciplinary program, highlighting how *co-production* can leverage social capital and inter-sectoral partnerships to enhance the capacity of marginalized urban communities to address broad health and environmental challenges through locally designed sustainable interventions (42). In the context of participatory research, particularly those in informal urban settlements, our program emphasizes several points that contribute to the research field.

Foremost, working simultaneously in three communities allowed integration of diverse knowledge, perspectives, and priorities, with a particular focus on engaging women and youth. Secondly, the communities identified priorities and targets based on their specific cultural, social, and environmental perspectives. Thirdly, the unique interventions implemented by each community to tackle similar problems demonstrated that optimal solutions are context-dependent rather than universally “best.”

Furthermore, from the outset, our team prepared to evaluate changes in incidence of specific infectious diseases and environmental indicators as metrics to accompany the interventions. Interestingly, the communities primarily chose multi-purpose interventions targeting general wellbeing—choices that differed from our expectations based solely on previously collected eco-epidemiological data (10). This unexpected preference highlighted the importance of addressing broader social and collective determinants of health and will allow us to test whether the benefits of these holistic interventions extend to more specific outcomes, such as reductions in single diseases or improvements in environmental indicators (43, 44). Another innovative aspect was implementing a feedback loop connecting the *activities* organized by our team and grounded in Popular Health Education (45) with the *interventions* initiated by the community. This dynamic facilitated continuous dialogue and mutual adaptation, enabling us to refine our activities and research instruments both within and across disciplines.

Below, we describe these dimensions in more detail and provide examples from the three communities where the program was implemented. We also describe how community participation led to setting joint agendas and plans with governmental agencies, in addition to secondary and medium- and long-term benefits for the community. Finally, we identify some of the challenges that our program faced and the lessons that can be learned for adapting such a program to other contexts.

### Unique solutions to common challenges

Our program embraced the diverse knowledge and perspectives both within and among communities, reflecting differences in demographic, socioeconomic, and cultural characteristics of existing organizations, newly formed community groups, and participating residents. Thus, while the three communities face similar health and quality of life challenges (Table 1), their distinct profiles have shaped local priorities, resources, and methods of engagement, leading to a diversity of solutions tailored to their specific needs (28). This aligns with earlier studies emphasizing that context-specific and inclusive interventions are more effective than one-size-fits-all models (46, 47). By remaining attentive to the unique journey of each community, including their distinct histories, starting points, and processes, we aim to understand, upon evaluating the interventions in the next phase of the program, how the local context can influence priorities, interventions, and their outcomes, which will aid adapting the program to other contexts nationally and internationally (48).

For example, the active participation of women and youth introduces diverse perspectives that are more attuned to the broader needs of the community (49). Despite their important role, these groups are frequently underrepresented in participatory initiatives, leading to gaps in addressing their specific needs and perspectives (50). Previous studies have reported improved health outcomes in community health initiatives where women's participation is strengthened (51, 52). However, in this study, women's increased participation is interpreted within the context of existing structural and social gender inequalities that often position women more prominently within community and household health-related activities, rather than as an indication of an inherent or exclusive responsibility for care. The program therefore sought to recognize and support female leadership while emphasizing shared, collective, and equitable responsibility among all community members in health promotion and decision-making (51, 52).Their involvement often results in interventions that prioritize social determinants of health and strengthen community ties and capacity. These include gender-specific health needs, youth engagement strategies, child welfare, cultural preservation, and innovative approaches to community development (49). Similarly, youth engagement ensures that interventions are aligned with the needs of younger generations (53). In Pau da Lima, for example, most participants were women. They highlighted social vulnerabilities related to gender, class, and ethnicity, such as food insecurity, violence, and mental health. These themes were closely linked to the interventions proposed, which included a community garden and kitchen, sports and artistic activities, and workshops (e.g., crafts and makeup).

### Evaluating broad interventions with a wide range of metrics

Rather than imposing predefined intervention targets, which often limits participation and local input in relation to what solutions are prioritized and how they are implemented (48, 54, 55), our program facilitated interventions that emerged organically from the concerns of the involved communities. The challenges identified spanned environmental, individual, and community levels, including environmental quality, infectious diseases, physical and mental wellbeing, food security, and violence, as well as less tangible elements like memory, identity, and sense of community. Interestingly, the interventions chosen in all three communities overwhelmingly targeted general wellbeing, which differed from what we might have selected as researchers relying solely on eco-epidemiological data.

From the outset, our program was prepared to continuously update existing evaluation metrics and introduce new ones. Thus, our approach to evaluating these complex interventions is to acknowledge that addressing broad, overarching wellbeing goals can have ripple effects on specific outcomes such as disease prevalence and environmental health (56). Hence, our program will define intervention success not only through individual-level outcomes (e.g., disease incidence) and at community, animal, and environmental levels, but also, more importantly, by fully appreciating community health and empowerment (54). This includes revealing whether interventions targeting individual and community wellbeing, shaped by their choices, provide specific health benefits, and challenges the assumption that the most effective interventions are those defined solely by a narrow set of predefined outcomes.

For example, in Alto do Cabrito, the decision to create a memorial to reclaim the identity and history of the community became a catalyst for further interventions, including reclaiming a dumpsite as a football field in the “Bota Fora” intervention which was also implemented in Marechal Rondon. This transformation is expected not only to enhance physical and mental health but also to reduce inadequate waste disposal, potentially decreasing rat presence and diminishing the transmission of leptospirosis—a key infectious disease in the community (10). In Pau da Lima, sports and creative workshops for children extended beyond traditional health concerns by addressing social integration and personal development. These projects spurred increased participation by youth and their mothers in broader health initiatives, leading to the creation of a community garden and kitchen that addressed food insecurity while providing environmental and economic benefits, potentially reducing disease risks (by removing resources for rats and mosquito breeding sites), and fostering community resilience.

### Interdisciplinarity of focus

Throughout the program, our research teams needed to adapt to multi-target and interlinked interventions by moving beyond narrow disciplinary boundaries and engaging in processes of shared learning with community participants. This academia–community articulation reflected principles commonly associated with university extension practices, characterized by reciprocal knowledge exchange and collective problem-solving between researchers and residents. Regular meetings and workshops involving community representatives were central to synthesizing diverse disciplinary and experiential perspectives, ensuring that insights generated within one group informed and enriched the methodologies and approaches of others. For example, when residents identified potential hotspots of disease transmission through collaborative mapping, the epidemiology and ecology teams adjusted their sampling strategies accordingly, while social scientists conducted in-depth interviews to better understand exposure risks and barriers to behavioral change. This iterative process strengthened both research design and community engagement by integrating local knowledge with scientific inquiry.

This feedback loop between *activities* and *interventions* culminated in interventions that were more creative and multi-targeted than what would have likely been proposed by a more conventional approach, driven mainly by formally trained researchers (57–60), or with an approach where both researchers and community cross-fertilized ideas but were limited within a fixed scope of intervention targets and outcomes (61, 62).

### Short and long-term benefits

In the short term, our program led to the design and implementation of interventions to address health concerns as perceived by the community residents. It has promoted, within marginalized communities, participation in local planning and governance (63), and the possibility of setting joint priorities and agendas with governmental agencies, with three hearings made between 2022 and 2024 on “Bota For a,” and “Living Green Dyke” interventions that required continuous collaboration with three agencies (LIMPURB, SEMOP, and INEMA). Furthermore, it has supported the establishment of common ground among different communities and the emergence of leaders from historically marginalized groups. These leaders play a crucial role in both the implementation and sustained maintenance of interventions. Our contention is that interventions in informal settlements may have significant public value (for example, through empowerment), even when their effects are not immediately measurable using conventional epidemiological or ecological metrics.

Nevertheless, in the longer term, in the next phase of the program, there will be a robust evaluation of its impacts, assessed not only through the interventions themselves, but also by evaluating their implementation (64). This distinction is vital for driving sustainable changes at local scales. When interventions fail to meet expectations, it is essential to identify whether the failure was due to the intervention being ineffective (intervention failure) or the improper execution of an otherwise effective intervention (implementation failure). Evaluating implementation effectiveness will involve assessing key factors such as acceptability, appropriateness, feasibility, uptake (adoption), reach (penetration), fidelity, cost, and sustainability (64).

### Challenges

The CBPR process faced significant resource and timing constraints. At the community level, several structural challenges affected implementation. Many communities lacked pre-existing representative groups and experienced fragile social cohesion, compounded by violence and insecurity. In Pau da Lima, the absence of shared community spaces limited opportunities for social interaction and collective organization, leading to the creation of the “Freirean Tent” activities. These activities later contributed to the formation of the United Community Group, a name chosen by participants to emphasize unity as a pathway toward improved rights, living conditions, and health outcomes. Physical space limitations also affected the implementation of activities such as boxing and theater workshops, reducing children's participation. Adverse weather conditions and insecurity, including police operations, further disrupted activities and reflected broader challenges related to social invisibility and incomplete realization of established social rights.

From the research perspective, building trust among interdisciplinary teams and community members, embracing the evolving nature of CBPR, and integrating diverse disciplinary approaches presented both challenges and opportunities for learning. Conducting participatory research during the COVID-19 pandemic added additional complexity, as interactions were restricted to virtual meetings for extended periods. Defining the scope of participation and ensuring meaningful co-construction of knowledge required continuous adaptation, leading the research team to expand its conceptualization of health in alignment with local realities and to repeatedly reassess research protocols and timelines. Although the diversity of methods and engagement strategies made it difficult to maintain a linear research trajectory, it fostered sustained dialogue among stakeholders and helped avoid the imposition of a single interpretative framework.

## Conclusion

This article describes a Community-Based Participatory Research (CBPR) approach, combined with principles of Popular Health Education, implemented to address health challenges in neglected urban communities in Salvador, Brazil. The approach was characterized by the active engagement of diverse community members and the incorporation of multiple perspectives shaped by gender, age, and social position. Rather than focusing exclusively on infectious diseases, the process enabled the identification of interconnected health concerns, including food security, violence, memory, and identity, reflecting broader social determinants of health. By integrating local knowledge with flexible and interdisciplinary research practices, the project supported the co-development of community-driven interventions aligned with locally identified priorities.

Despite challenges related to limited resources, insecurity, and the constraints imposed by the COVID-19 pandemic, the participatory process contributed to strengthening community dialogue, fostering collective action, and supporting the emergence of local leadership, particularly among groups historically underrepresented in decision-making spaces. The experience highlights the importance of sustained academia–community collaboration and adaptive research processes when working in contexts marked by social vulnerability. Further research is needed to evaluate the short- and long-term impacts of these interventions and to assess their potential sustainability and transferability to similar urban settings.

## Data Availability

The datasets presented in this article are not readily available because all relevant data used in this manuscript are included in the form of tables or text descriptions. Additional datasets generated through this project can be provided upon reasonable request from the Data Manager at nivisonjr@gmail.com. Requests to access the datasets should be directed to nivisonjr@gmail.com.
